# Ultra short term power load forecasting based on the fusion of Seq2Seq BiLSTM and multi head attention mechanism

**DOI:** 10.1371/journal.pone.0299632

**Published:** 2024-03-22

**Authors:** Yuanfang Gou, Cheng Guo, Risheng Qin

**Affiliations:** 1 School of Mechanical and Electrical Engineering, Kunming University of Science and Technology, Kunming, Yunnan, China; 2 School of Electric Power Engineering, Kunming University of Science and Technology, Kunming, Yunnan, China; 3 Yunnan Power Grid Company Limited Electric Power Research Institute, Kunming, Yunnan, China; Abu Dhabi University, UNITED ARAB EMIRATES

## Abstract

Ultra-short-term power load forecasting is beneficial to improve the economic efficiency of power systems and ensure the safe and stable operation of power grids. As the volatility and randomness of loads in power systems, make it difficult to achieve accurate and reliable power load forecasting, a sequence-to-sequence based learning framework is proposed to learn feature information in different dimensions synchronously. Convolutional Neural Networks(CNN) Combined with Bidirectional Long Short Term Memory(BiLSTM) Networks is constructed in the encoder to extract the correlated timing features embedded in external factors affecting power loads. The parallel BiLSTM network is constructed in the decoder to mine the power load timing information in different regions separately. The multi-headed attention mechanism is introduced to fuse the BiLSTM hidden layer state information in different components to further highlight the key information representation. The load forecastion results in different regions are output through the fully connected layer. The model proposed in this paper has the advantage of high forecastion accuracy through the example analysis of real power load data.

## 1 Introduction

The demand for electricity is growing with the continuous development of the socio-economic level [1]. Accurate load forecasting becomes more and more important as in the background of spot market, the contracting strategy, quotation strategy and trading strategy of power sales companies are based on the results of load forecasting, which not only helps power supply enterprises adjust their power generation plans in a timely and economical manner, but also helps ensure the safe and stable operation of the power system [2]. Therefore, the forecasting of ultra short term power load has been the focus of extensive attention of industry and academia.

Presently, a substantial amount of research has been devoted by domestic and international scholars to the precise prediction of ultra-short-term power load. This research can be broadly categorized, in terms of the model’s complexity, into two approaches: mathematical statistics methods and machine learning methods. Among these, mathematical statistical models mainly comprise multi-linear regression [3, 4], the Carlman filter model [5], and the time sequence method [6, 7]. These models require small data volumes, high data correlation demands, and some algorithms require expert experience, which is not able to accurately express the variable factors affecting the load. Traditional machine learning models such as random forest [8] and support vector machine [9, 10] and decision tree [11] are widely used to explore nonlinear mapping relationships between power loads. Sun Hairong et al. [12] proposed a predictive model based on particle swarm optimization support vector machine parameters to realize the prediction of short term power load according to the time-varying and nonlinear characteristics of power load. Chauhan M et al. [13] proposed a short-term power load forecasting model construction method based on support vector machine and integrated learning idea, which improved the accuracy of power load forecasting. DANG S et al. [14] proposed a short-term load forecasting method based on random forest combined with quantile regression to quantify the uncertainty of power load. These methods can effectively predict the power load to a certain extent, but the power system load forecasting model is a high-dimensional nonlinear complex system, which contains a variety of data flows such as power flow, weather flow and information flow [15]. These data information blend with each other, traditional machine learning is difficult to fully learn and express the internal correlation time series information, and it needs to rely on the method of deep learning to deeply mine the nonlinear relationship between power load and external factors such as temperature, humidity and time attributes. For example, the recurrent neural network [16, 17] uses the network structure of cyclic feedback to recursively replace time sequence information in accordance with the direction of sequence evolution. Ren Jianji et al. [18] used a combination of CNN and BiLSTM network to fully extract the potential spatio-temporal characteristics of load data, and introduced the attention mechanism to automatically distinguish the Importance degree of different time attributes. Yang Shuqiang et al. [19] proposed a method of graphic power load data and using Long short-term memory (LSTM) network to mine time series load data for short-term power load prediction, so as to improve the accuracy of power load prediction. Yan Hong et al. [20] used time convolution network (TCN) and gate recurrent unit (GRU) to fully extract the potential spatio-temporal characteristics of load data, and introduced attention mechanism to automatically distinguish the importance of different time load sequences. Masood Z et al. [21] used the sequence to sequence learning framework to introduce the LSTM network as the encoding and decoding network for constructing a multi-step load sequence learning model. Khan Z A et al. [22] designed the deep residual convolutional neural network to extract the important features affecting the load, and in series stacked the LSTM network to learn the time information of power data. Mu Y et al. [23] achieved an accurate prediction of power load by combining the sequence-to-sequence structure with the LSTM model, which well reflected the time sequence dependence between output tags.

The above-mentioned in-depth learning method has yielded numerous positive outcomes, but there are still the following shortcomings for ultra-short-term power load forecasting scenarios in different regions: 1) The influence of multi-dimensional data in different regions on power load is different, making it difficult to characterize the time sequence correlation information between the data. 2) Power load forecasting is dependent not only on historical load data but also on the effective information between other load data.

Based on the above analysis, aiming at the complex nonlinearity and time series characteristics of power load, this paper proposes a hybrid neural network ultra short term power load forecasting method based on sequence to sequence framework. It aims to learn the correlation time series relationship between different dimensional data streams in the power system and the potential temporal information of the power load simultaneously with the help of sequence-to-sequence learning structure. The CNN uses convolution kernel to extract effective nonlinear local features from power load data, and the pooling layer selects the maximum pooling method to compress the extracted features and generate more critical feature information. The BiLSTM hidden layer learns the internal dynamic changes of the local features extracted by CNN, and iteratively extracts more intricate global features from the local features. On this basis, the features generated by the BiLSTM hidden layer are used as the input of the attention mechanism. The attention mechanism is used to automatically allocate the corresponding weight to the time information extracted by the BiLSTM hidden layer, distinguish the importance of different time load series, highlight the information of key historical time points, and reduce the impact of redundant information on the load forecasting results. Finally, the study utilizes the actual power load data from Tétouan, Morocco to demonstrate the effectiveness of the proposed CD_Bilstm model in ultra short-term load forecasting. Four different scenarios of power load forecasting are conducted based on the power data from four quarters. These scenarios are then compared with four recent forecasting methods to validate the superior performance of the CD_Bilstm model, which combines convolutional neural network with multi head attention mechanism parallel bidirectional long-term and short-term memory network. The contributions of this paper are as follows:

This paper mainly realizes the comprehensive mining and forecasting of ultra short term load from two aspects: the extraction of internal dynamic change rules of in second put characteristics and the optimization of forecasting model.

1. In terms of input characteristics extraction, CNN uses convolution kernel to extract effective nonlinear local features from power load data, and the pooling layer selects the maximum pooling method to compress the extracted features and generate more critical feature information, thereby mining the load’s inherent regularity and enhancing generalization ability.
2. In terms of prediction model optimization, BiLSTM takes into account both past and future information. By focusing on the forward sequence information input and the backward sequence information input, Bi LSTM extracts the bidirectional time series characteristics of sequence data, which is conducive to further improving the accuracy of model prediction. At the same time, the attention mechanism is used to quantify the correlation between input characteristic variables, capture the dependence of time sequence information, enhance the information expression of significant characteristic variables and crucial time steps, highlight the information of key historical time points, mitigate the influence of miscellaneous information on load forecasting results, and improve the time sequence forecasting ability of the algorithm.

## 2 Materials and methods

### 2.1 Model structure

**Fig 1:**
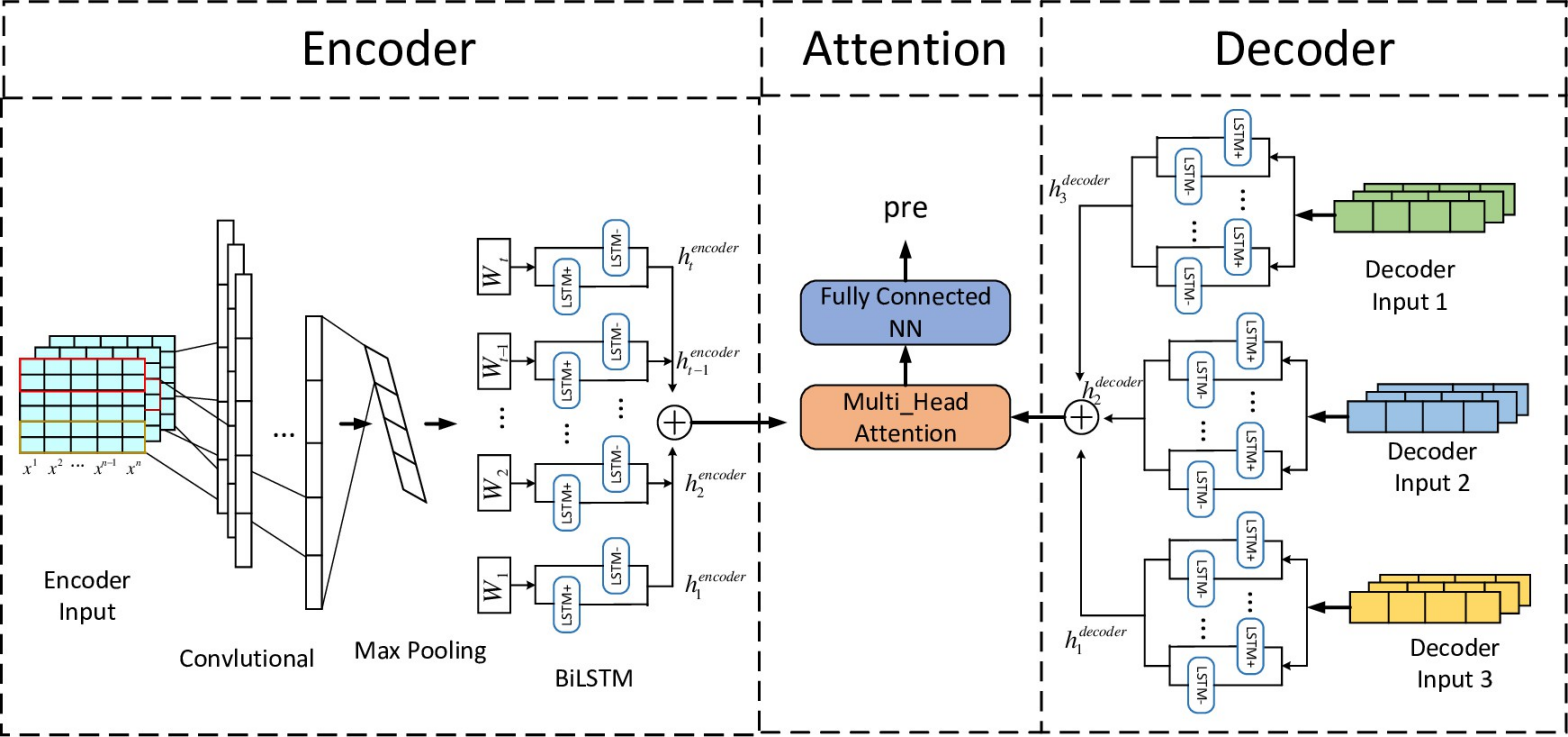
CD_BiLSTM network overall structure.

With the help of sequence-to-sequence learning structure, the model simultaneously extracts the exogenous features of power load and its own time-series features, and deeply learns the power load related information to realize the ultra-short-term power load forecastion. As shown in Fig 1, the hybrid network model constructed in this paper is based on the sequence-to-sequence framework, which comprises an encoder and decoder. The encoder part constructs the CNN and BiLSTM combined network, uses the power load data as the input data of the coding component, uses the convolution and pooling layer in CNN to acquire potential depth information regarding external factors that impact power load, transforms this information into fixed length vector storage and applies BiLSTM network bidirectional iterative processing to the time sequence data, guarantees that the state variables of the coding layer contain the temporal correlation information of objective factors at each time. The decoder part uses parallel BiLSTM to achieve long-term correlation information extraction of power loads in different regions ensuring that the long-term equilibrium relationship between power load data in different regions be remained. On this basis, the multi head attention mechanism is embedded to efficiently integrate all feature information, pay attention to the features extracted by different components, focus on the key information, and avoid the interference of redundant information, and finally take different regional power load sequences in subsequent time as the target output value.

### 2.2 Encoder based on CNN-BiLSTM feature learning

Deep learning is an effective method to characterize the correlations between power load related features compared with traditional power load forecasting models. Due to the characteristics of periodicity and time variability of power load fluctuations, it is necessary to deeply mine the time characteristics and correlation relationship between the historical data of power load and external factors, and the real-time order correlation relationship. Therefore, this paper introduces the feature learning ability of convolutional neural network into the encoder, and extracts the bidirectional timing dependence characteristics in series with BiLSTM, and comprehensively applies CNN-BiLSTM as the encoder component.

CNN has the ability to automatically learn the potential characteristics of depth, and can effectively get rid of the dependence on the correlation parameters obtained by expert experience. It primarily relies on the convolution and pooling structure implemented in CNN, uses the sliding window operation of convolution kernel to capture the static characteristics of time-series data, and then uses the scale invariance of the key features in the pooling layer to reduce the dimension of the extracted features, highlight the key features, and use parameter sharing to reduce the complexity of the network.

The feature mapping obtained for the *m*th objective factor in moment *t* is $F_{m}^{t}$, whose vector dimension is *H*_*t*_ × *W*_*t*_ × *C* × *s*. *H*_*t*_, *W*_*t*_ and *C* are the height, width and channel of the feature map respectively. *s* is the time step. *N* is the total number of external factors, as shown in Eq (1), the total power load time series feature mapping set is:

$$F_{CNN}=\sum_{m=1}^{N}F_{m}^{t}(x,y)$$ (1)

**Fig 2:**
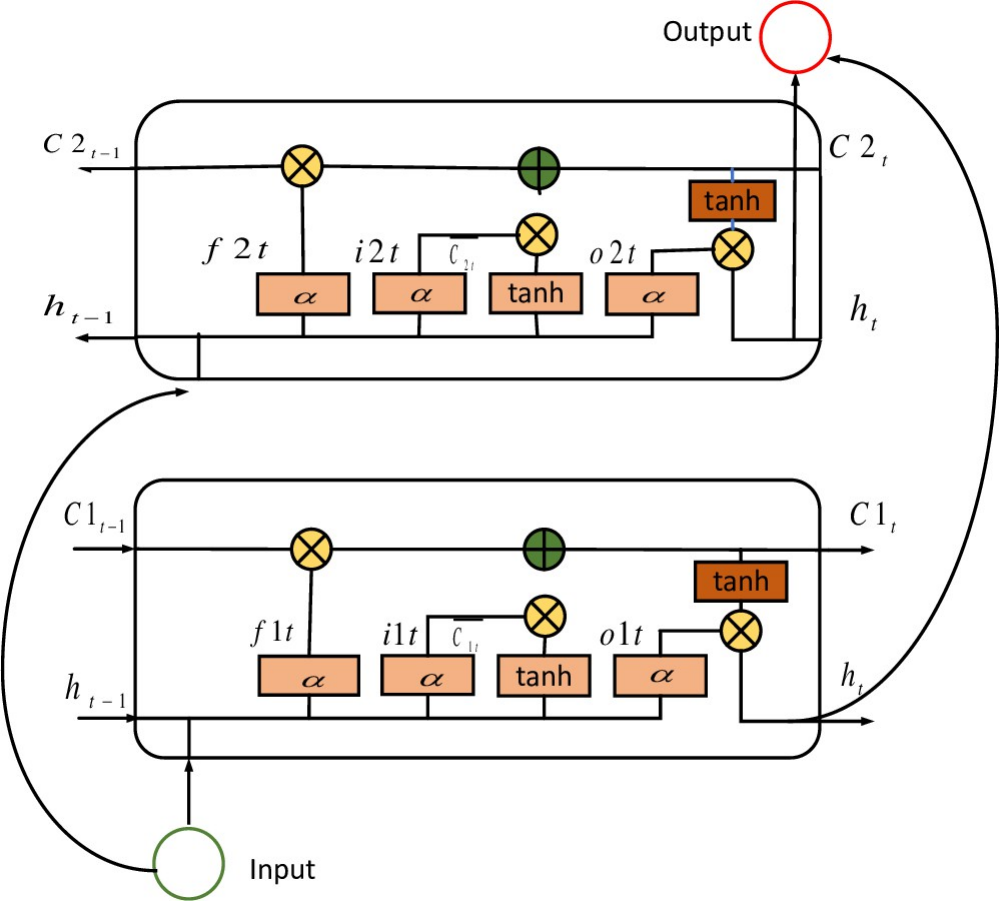
BiLSTM network structure.

For the complex temporal correlation information in the actual consumption process of electric load, firstly, CNN is used to construct the objective factor matrix of electric load into a time-domain feature information matrix according to certain rules. Then, the temporal characteristics in the time-domain matrix are extracted by recurrent neural network learning and defined as a fixed-length vector. BiLSTM is an extended structure based on the evolution of long and short term memory network, the core idea is to add another layer of LSTM on top of the original LSTM to reverse process the data, whose structure is shown in Fig 2. BiLSTM uses two independent LSTMs to mine the process timing information from the front and back ends, which can completely capture the bi-directional time structure information in multi-sequence data.

The lower layer is a forward LSTM and the upper layer is a backward LSTM, both using the same computational process. LSTM uses a gated output approach, i.e., input gate, forget gate, output gate and two temporal states (Cell State and Hidden State). The output values at moment *t* are *i*_*t*_, *f*_*t*_, *o*_*t*_, *c*_*t*_ and *h*_*t*_, respectively, and are solved in Eq (2):

$$\left\{\begin{array}{l} i_{t}=\sigma (W_{xi}x_{t}+W_{hi}h_{t-1}+W_{ci}c_{t-1}+b_{i})\\ f_{i}=\sigma (W_{xf}x_{t}+W_{hf}h_{t-1}+W_{cf}c_{t-1}+b_{f})\\ o_{t}=\sigma (W_{xo}x_{t}+W_{ho}h_{t-1}+W_{co}c_{t-1}+b_{o})\\ c_{t}=f_{t}⊙c_{t-1}+i_{t}⊙\text{tanh}(W_{xc}x_{t}+W_{hc}h_{t-1}+b_{c})\\ h_{t}=o_{t}⊙\text{tan}(c_{i}) \end{array}\right.$$ (2)

The given input sequence is *x* = {*x*_1_, *x*_2_, *x*_3_, ⋯, *x*_*t*_, ⋯, *x*_*T*_}, where *t* denotes the *t* moment and *T* denotes the total number of time, and finally the output result is obtained in Eq (3).

$$\left\{\begin{array}{l} h_{t}=\sigma (W_{xh}x_{t}+W_{hh}x_{t-1}+b_{h})\\ \hat{x}=\sigma (W_{ho}h_{t}+b_{o}) \end{array}\right.$$ (3)

In the above equation: *h*_*t*_ denotes the output of the hidden layer at time *t*. ${\overset{⌢}{x}}_{t}$ denotes the LSTM output result at time *t*. *σ* is the Sigmids activation function. *b*_*α*_ denotes the deviation, where *α* ∈ {*i*, *f*, *c*, *o*, *h*}, W = {*W*_*xi*_, *W*_*hi*_, *W*_*ci*_, *W*_*xf*_, *W*_*hf*_, *W*_*cf*_, *W*_*xo*_, *W*_*ho*_, *W*_*co*_, *W*_*xc*_, *W*_*hc*_, *W*_*xh*_, *W*_*hh*_} denotes the weighting parameter obtained by time back-propagation, such as the weight matrix between the input layer and the hidden layer.

BiLSTM, on the other hand, obtains the final output value by stitching the output of two LSTM layers in Eq (4):

$$\left\{\begin{array}{l} h_{t}=\alpha h_{t}^{f}+\beta h_{t}^{b}\\ \hat{y}=\sigma (h_{t}) \end{array}\right.$$ (4)

where: $h_{t}^{f}$ and $h_{t}^{b}$ are the outputs of the forward and backward LSTMs, respectively, with opposite sequence order as input. *α* and *β* are the factors of the sequence control forward and backward LSTM(*α* + *β* = 1). *h*_*t*_ denotes the output value of the hidden layer state of the BiLSTM at time *t*. ${\overset{⌢}{y}}_{t}$ is the output value of the overall BiLSTM.

To obtain complete feature information, the encoder in this model uses CNN_BiLSTM to achieve autonomous feature learning and express potential deep information, which extracting complex time-series correlation information embedded in external factors affecting power load. First, the objective factor data is used as input data for the coding component, which is fed into the convolutional neural network to automatically extract the feature mapping vector *F*_*CNN*_, and then fed into the BiLSTM network to iterate the timing information in both directions, ensuring that the state variables of the coding layer at each moment contain the timing association information of the objective factors.

### 2.3 Decoder incorporating multi-headed attention mechanism in parallel with BiLSTM

**Fig 3:**
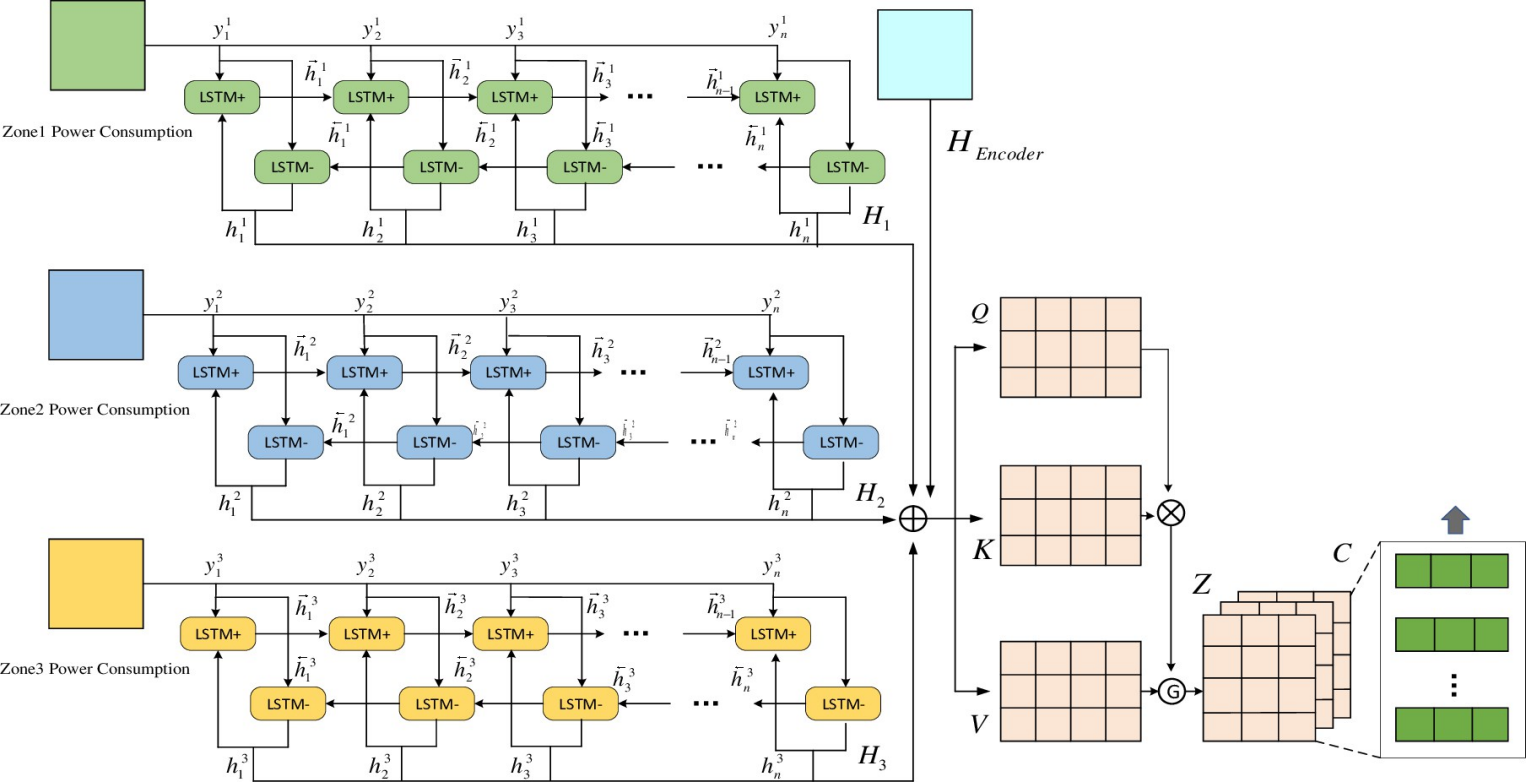
Decoder network structure.

Considering the long-term equilibrium relationship between the electric loads in different regions, as shown in Fig 3, the parallel network model is used to process the source data of electric loads in each region separately. And the time series data from a single source are learned independently using the BiLSTM network to obtain the time dependence between the explanatory and explained variables from each source. Further more, the multi-headed attention mechanism is used to synthesize the information of each module feature, including the coding layer information, allowing the model to jointly focus on the information from different representation subspaces in different locations.

Here is obvious time-series information, as the power load data of each region changes with time. BiLSTM is used to model the power load sequence and extract the bi-directional time-series information from the data, learning the cell information of each moment forward and backward to obtain the output values ${\vec{h}}_{j}^{i}\in R^{N\times p}$ and ${\overset{\leftarrow }{h}}_{j}^{i}\in R^{N\times p}$ at each time step, as shown in Eqs (5) and (6). The parallel network model can be designed to learn independently for a single data source, avoiding the association of nodes between different data sources and reducing the consumption of computing power resources. The individual regional power load data are selected and input to the BiLSTM network, and the outputs of the forward and backward networks are obtained accordingly, and the integrated information is calculated to obtain $h_{j}^{i}$, *i* ∈ {1, 2, 3}, as shown in Eq (7). And then the two-way timing information from independent data sources is aggregated *H*_*i*_ ∈ *R*^*N*×2*p*^.

$${\vec{h}}_{j}^{i}=\vec{LSTM}(e_{j}^{it},{\vec{h}}_{j}^{i(t-1)};W_{j}^{decoder})$$ (5)

$${\overset{\leftarrow }{h}}_{j}^{i}=\overset{\leftarrow }{LSTM}(e_{j}^{it},{\overset{\leftarrow }{h}}_{j}^{i(t-1)};W_{j}^{decoder})$$ (6)

$$H_{i}=(h_{1}^{i},h_{2}^{i},h_{2}^{i},\cdots ,h_{t}^{i})$$ (7)

Where: $e_{j}^{t}$ is the power load in region *j* at moment *t*. $h_{j}^{t-1}$ is the hidden layer state of the LSTM at moment *t-1*. $W_{j}^{decoder}$ is the weight matrix of the *j*th BiLSTM.

**Fig 4:**
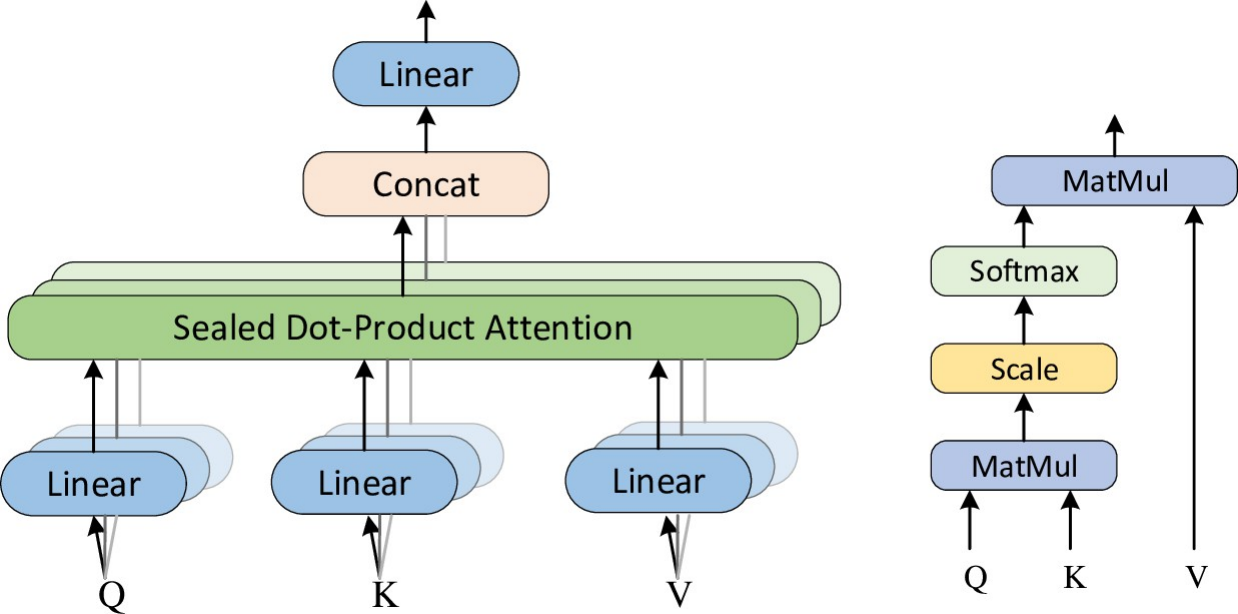
Multi-headed attention structure.

Information fusion is achieved using multi-headed attention networks to integrate parallel BiLSTM network power load timing information in the redistribution decoder and CNN_BiLSTM network timing association information in the encoder. This mechanism uses multiple independent attention functions to integrate information from different subspaces and enhance the feature representation of the forecasted target. The multi-headed attention mechanism aggregates the encoder implicit state information *H*_*encoder*_ and the decoder parallel BiLSTM state matrix as input, and transforms the input matrix into three matrices of the same dimension *Q*, *K*, *V* ∈ *R*^*N*×*d*^ by linear transformation, as shown in Eqs (8)–(10). The network structure of the multi head attention mechanism is shown in Fig 4.

$$\begin{array}{l} M^{t}=Agg(H^{encoder},H_{j}^{i})\\ =\{Agg(H_{1}^{encoder},\sum_{i=1}^{n=3}H_{j1}^{i}),\cdots ,Agg(H_{N}^{encoder},\sum_{i=1}^{n=3}H_{jN}^{i})\} \end{array}$$ (8)

$$Q^{m}=M^{t}W^{q,m}$$ (9)

$$K^{m}=M^{t}W^{k,m}$$ (10)

$$V^{m}=M^{t}W^{v,m}$$ (11)

Where *Agg* denotes the summation of the temporal correlation matrix of the power load-related factors at time *t* with the corresponding elements of the power load temporal state matrix. And *M*^*t*^ ∈ *R*^*t*×*d*^ is the complete matrix obtained after information aggregation. *W*^*q*,*m*^, *W*^*k*,*m*^, *W*^*v*,*m*^ ∈ *R*^*d*×*d*^ are the transfer matrices corresponding to *Q*^*m*^, *K*^*m*^, *V*^*m*^ under *m*(*m* ∈ {1, ⋯, *l*} linear transformations, respectively. *l* linear transformations are able to capture the correlation roles between external factors of power load, historical power load and power load at future moments in *l* different perspectives, as shown in Eq (12). *Q*^*m*^, *K*^*m*^, *V*^*m*^ are firstly scaled dot product attention calculation to obtain the attention context vector of the *i*th one.

$$head^{m}=Attention(Q^{m},K^{m},V^{m})=softmax(\frac{Q^{m}•K^{m}}{\sqrt{d}})$$ (12)

Where: softmax function calculates the multi-source feature weights in *K*^*m*^ based on *Q*^*m*^, and *d* is the vector dimension.

The transfer matrices corresponding to *Q*^*m*^, *K*^*m*^, *V*^*m*^ are stitched into *l* attention context vector matrices by aggregating the degree of association of features under multiple attentions through linear transformation, as shown in Eq (13).

$$MHA^{m}(Q,K,V)=Concat(head^{1},\cdots ,head^{l})•W^{0}$$ (13)

*MHA*^*m*^ denotes the multi-source feature interaction state matrix at moment *t* obtained by the multi-headed attention mechanism, Concat denotes the connection operation, and *W*_0_ ∈ *R*^*ld*×*d*^ is the linear transformation matrix.

Before the final decoding, the power load value $\tilde{y}$ at the future time is obtained using the fully connected network, by splicing the multi-source time series correlation vector *M*_*i*_ obtained from different networks with the correlation degree information *MHA* obtained from the multi-headed attention mechanism. The calculation of $\tilde{y}$ value is shown in Eq (14).

$$\tilde{y}=FC([M^{l},MHA^{l}];W_{c})$$ (14)

Where: *FC* is the fully connected layer and *W*_*c*_ is the fully connected layer weight matrix.

## 3 Results and discussion

### 3.1 Experimental data acquisition and pre-processing

**Fig 5:**
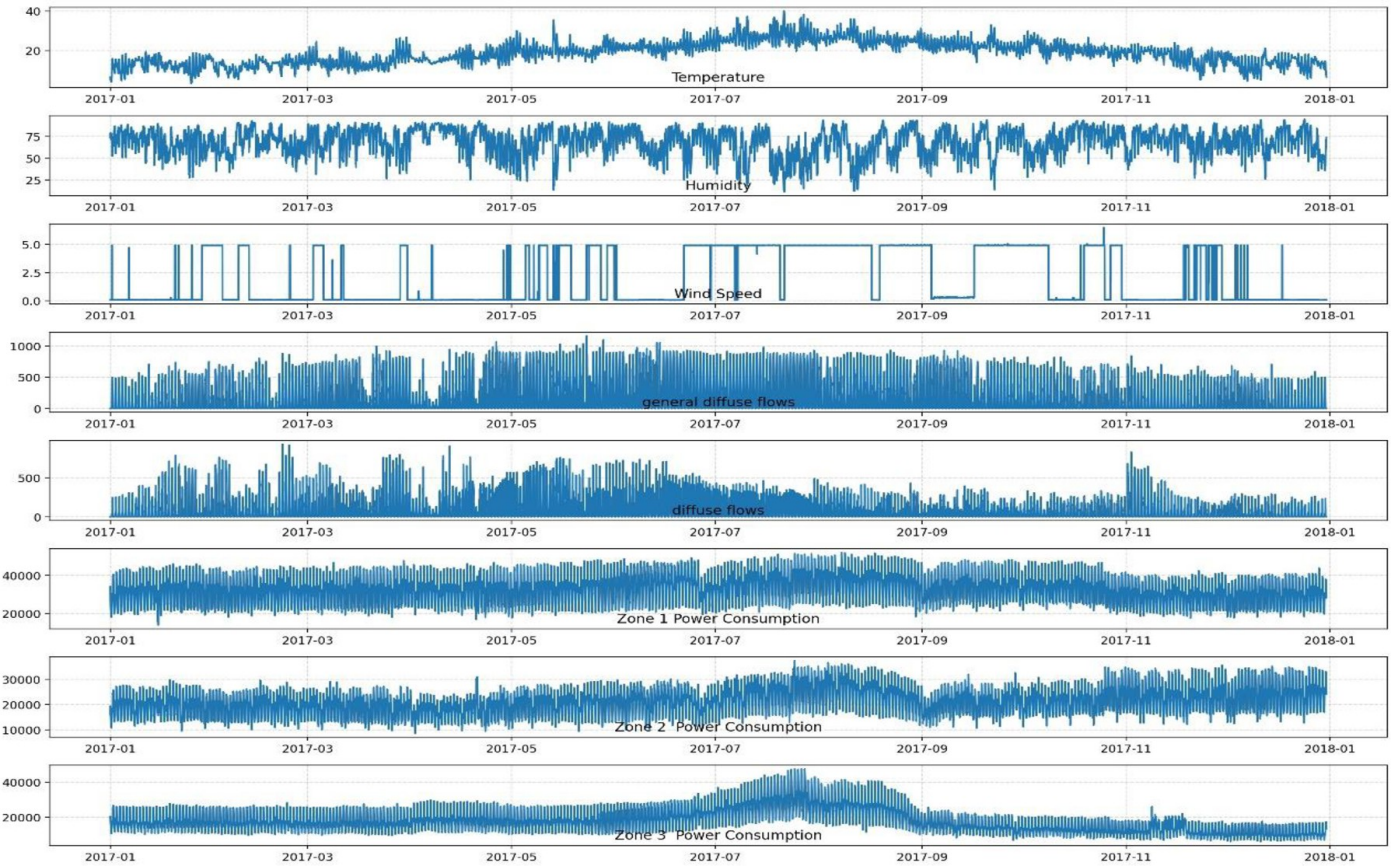
Changes in power load data.

**Fig 6:**
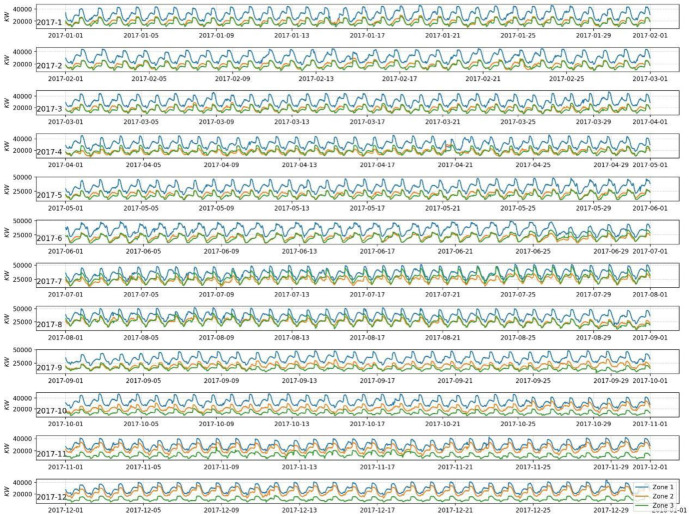
Monthly power load data changes for different regions.

To better illustrate the data acquisition and pre-processing methods, this paper uses practical electricity load data for the city of Tétouan, Morocco, for a total of 365 days from January 1, 2017 to December 30, 2017. This power load data includes multidimensional data of temperature, humidity, wind speed, general diffusive flow, diffuse flow and power load values of three different zones (Zone 1, Zone 2, Zone 3). The data are collected every 10 min, 144 points are collected per day. There are totally 52,416 electric load data, as shown in Fig 5. It can be easily seen from Fig 6 that the range distribution of electricity load under different months can be observed in more detail, with a significant increase in electricity consumption from May to September. As the data volatility will affect the prediction results, the data are accordingly divided into four phases by quarter and the power load prediction model is trained in different quarters. As shown in Table 1, the practical serial data are sequentially sliced according to the ratio of 8:2.

**Table 1:** Datasets divide information.

Dataset	Training data	Test Data
**First Quarter**	10368 Groups (2017-01-01-2017-03-13)	2592 Groups (2017-03-14-2017-03-31)
**Second quarter**	10483 Groups (2017-04-01-2017-06-12)	2621 Groups (2017-06-13-2017-06-30)
**Third Quarter**	10598 Groups (2017-07-01-2017-09-12)	2650 Groups (2017-09-13-2017-09-30)
**Fourth Quarter**	10483 Groups (2017-10-01-2017-12-12)	2621 Groups (2017-12-13-2017-12-31)

In the collected multidimensional data of electric load, since each parameter possess different dimension and level, it is necessary to normalize the data to control all indicators in the range of [0, 1]. The normalization process equation is as shown in Eq (15).

$$X_{i}=\frac{X_{i}-X_{\text{min}}}{X_{\text{max}}-X_{\text{min}}}$$ (15)

*X*_min_ is the minimum value in a single parameter. *X*_max_ is the maximum value in a single parameter.

### 3.2 Experimental setup

Network training and optimization were performed on a Dell DESKTOP-VI4SR6N server with an Intel(R) Core(TM) i7-8650U processor, 64GB of RAM, and UHD Graphics. The network is written in Python. And the CB_A_L neural network model is constructed in the Keras deep learning framework with Tensorflow as the backend. And the model is trained by the loss function and Adam optimizer.

The mean square error is applied to calculate the error loss, and the model training stops when the emor attenuation tends to stabilize, as shown in Eq (16)).

$$F_{loss}=\frac{1}{n}\sum_{i=1}^{n}{\left(y_{i}-{\hat{y}}_{i}\right)}^{2}$$ (16)

In addition, the MAE and RMSE are used as indicators to assess the predictive performance of the model. The error indicator is calculated in Eqs (17) and (18):

$$MAE=\frac{1}{n}\sum_{i=1}^{n}\left|y_{i}-{\hat{y}}_{i}\right|$$ (17)

$$RMSE=\sqrt{\frac{1}{n}\sum_{i=1}^{n}{\left(y_{i}-{\hat{y}}_{i}\right)}^{2}}$$ (18)

The goodness-of-fit (*R*_2_) is applied to judge the model fitting effect, which is caculated according to the Eq (19):

$$R_{2}=1-\frac{\sum_{i}^{n}{\left(y_{i}-{\hat{y}}_{i}\right)}^{2}}{\sum_{i}^{n}{\left({\bar{y}}_{i}-y_{i}\right)}^{2}}$$ (19)

Where: *y*_*i*_ is the true value of the electric load in each region at a certain time. ${\hat{y}}_{i}$ is the output value of the model, and ${\bar{y}}_{i}$ is the average value of the electric load in 3 regions at corresponding.

The parameters in the hybrid neural network prediction model based on the sequence-to-sequence framework are specifically set as follows: time step *s* = 144, sliding window *l* = 6, i.e., the historical electric load data of the previous day at a certain moment is used to predict the electric load value for the next one hour at that moment. The number of iterations is 1000. Learning rate = 0.001. The number of BiLSTM network layers in the model is 3, but the number of hidden layers of BiLSTM network in the encoder and decoder are different, *p* = 32 and *q* = 64 respectively.

The parameter setting of the CNN network in the encoder mainly adjusts the number of filters, and the rest of the parameters are fixed. For example, the default number of layers of the neural network is set to 1, the convolution step is set to 1, relu function is selected as the activation function, the electric load data of the first quarter is selected as an example to compare the results with different convolutional kernel comparison shown in Table 2.

**Table 2:** Comparison results of convolutional kernels of different CNNs.

Convolution kernel	Zone 1			Zone 2			Zone 3		
Size	MAE	RMSE	R2	MAE	RMSE	R2	MAE	RMSE	R2
**32**	721.11	1006.03	97.77	523.49	735.88	96.91	529.93	710.93	97.24
**64**	**707.69**	**970.32**	**98.03**	**498.68**	**660.25**	**97.48**	**431.22**	**584.89**	**98.05**
**96**	781.95	1055.86	97.49	505.78	673.55	97.24	491.68	650.62	97.50
**128**	798.61	1086.78	97.50	574.39	751.44	96.82	459.74	612.03	97.92
**256**	812.33	1256.98	96.87	587.45	873.22	95.78	672.47	796.38	95.48

As can be seen from the table, under the same conditions of other variables, the optimal size of CNN convolutional kernel in the CD_BiLSTM model constructed in the first quarter of this paper is 64, when the load prediction accuracy reaches the optimal state, considering the effect of electricity load prediction in different regions comprehensively.

### 3.3 Comparison experiment

To verify the prediction effectiveness of the model, the CD_BiLSTM model is compared with the methods that have shown better performance in power load forecasting in the past two years, the model mainly includes four models: Empirical Mode Decomposition and Extreme Learning Machine(EMD-ELM) [24], Empirical Mode Decomposition and Bidirectional Long Short Term Memory Network(EMD-BiLSTM) [25], Variational Modal Decomposition, Temporal Convolutional Network and Gated Recurrent Unit(VMD-TCN-GRU) [26], Temporal Convolutional Network, Gated Recurrent Unit and attention(TCN-GRU-Attention) [20]. The *MAE* and *RMSE* are selected as the model evaluation indexes, the results are shown in Table 3.

**Table 3:** Comparison of forecasting effects of different models in different quarters.

Dataset	Models	zone 1		zone 2		zone 3	
		MAE	RMSE	MAE	RMSE	MAE	RMSE
**First Quarter**	EMD-ELM	1727.3	2247.7	1732.0	2243.8	1492.4	2088.1
	EMD-BiLSTM	1501.7	1953.4	1346.1	1834.3	1351.8	1732.2
	VMD-TCN-GRU	1328.9	1747.0	1046.1	1407.5	949.3	1275.3
	TCN-GRU-Attention	894.8	1212.9	550.6	732.2	472.2	626.9
	**CD_BiLSTM**	**707.7**	**970.3**	**498.7**	**660.3**	**431.2**	**584.9**
**Second quarter**	EMD-ELM	3313.0	4501.3	2982.5	3533.9	2536.2	3308.5
	EMD-BiLSTM	2488.3	3137.0	2330.0	2896.5	2592.3	3172.6
	VMD-TCN-GRU	2355.3	3188.0	1588.6	1955.4	2350.2	3175.6
	TCN-GRU-Attention	1349.1	1675.2	760.56	937.9	1120.8	1507.2
	**CD_BiLSTM**	**1247.6**	**1637.2**	**666.2**	**921.5**	**1163**	**1419.1**
**Third Quarter**	EMD-ELM	3306.8	4184.5	1995.5	2607.5	4401.7	5637.8
	EMD-BiLSTM	2684.6	3625.6	1927.9	2542.8	4042.1	5386.4
	VMD-TCN-GRU	2663.4	3279.3	1282.0	1756.9	3815.5	5071.2
	TCN-GRU-Attention	878.7	1118.7	685.4	875.1	877.2	1138.2
	**CD_BiLSTM**	**730.7**	**1023.7**	**551.1**	**751.6**	**617.3**	**829.7**
**Fourth Quarter**	EMD-ELM	1860.1	2404.5	2411.4	2840.4	1457.0	1769.1
	EMD-BiLSTM	1389.2	1826.8	1220.8	1565.7	1265.8	1541.9
	VMD-TCN-GRU	1220.7	1620.3	1146.4	1464.1	1224.7	1528.8
	TCN-GRU-Attention	881.5	1132.8	627.6	808.0	526.8	648.8
	**CD_BiLSTM**	**589.9**	**751.2**	**416.2**	**552.0**	**322.2**	**428.4**

Table 3 demonstrates that our model outperforms other methods in terms of *MAE* and *RMSE* values, demonstrating superior predictive performance and stability in our predictions. The CD_BiLSTM model exhibits a notable decrease in *MAE* and *RMSE* values when compared to shallow learning models such as EMD-ELM and EMD-BiLSTM. This indicates that the CD_BiLSTM model is proficient at extracting temporal information from the data. In contrast to deep learning combination models such as VMD-TCN-GRU and TCN-GRU Attention, the model described in this article employs a complex deep learning structure to effectively capture deep temporal correlation features in power load data. This results in reduced *MAE* and *RMSE* values, as well as improved prediction accuracy and efficiency of the model.

**Fig 7:**
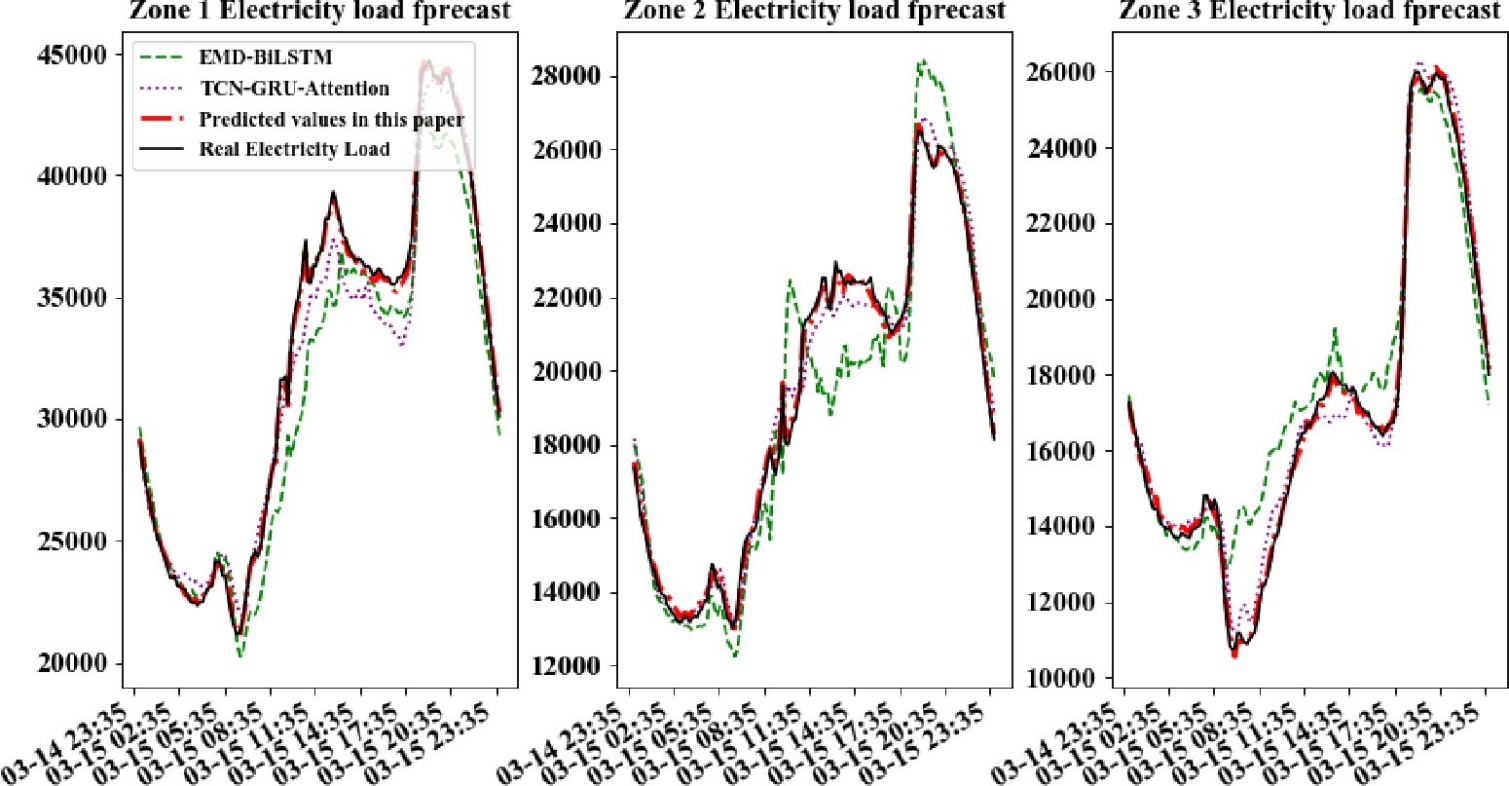
Comparison of forecast model results for a day in the first quarter.

**Fig 8:**
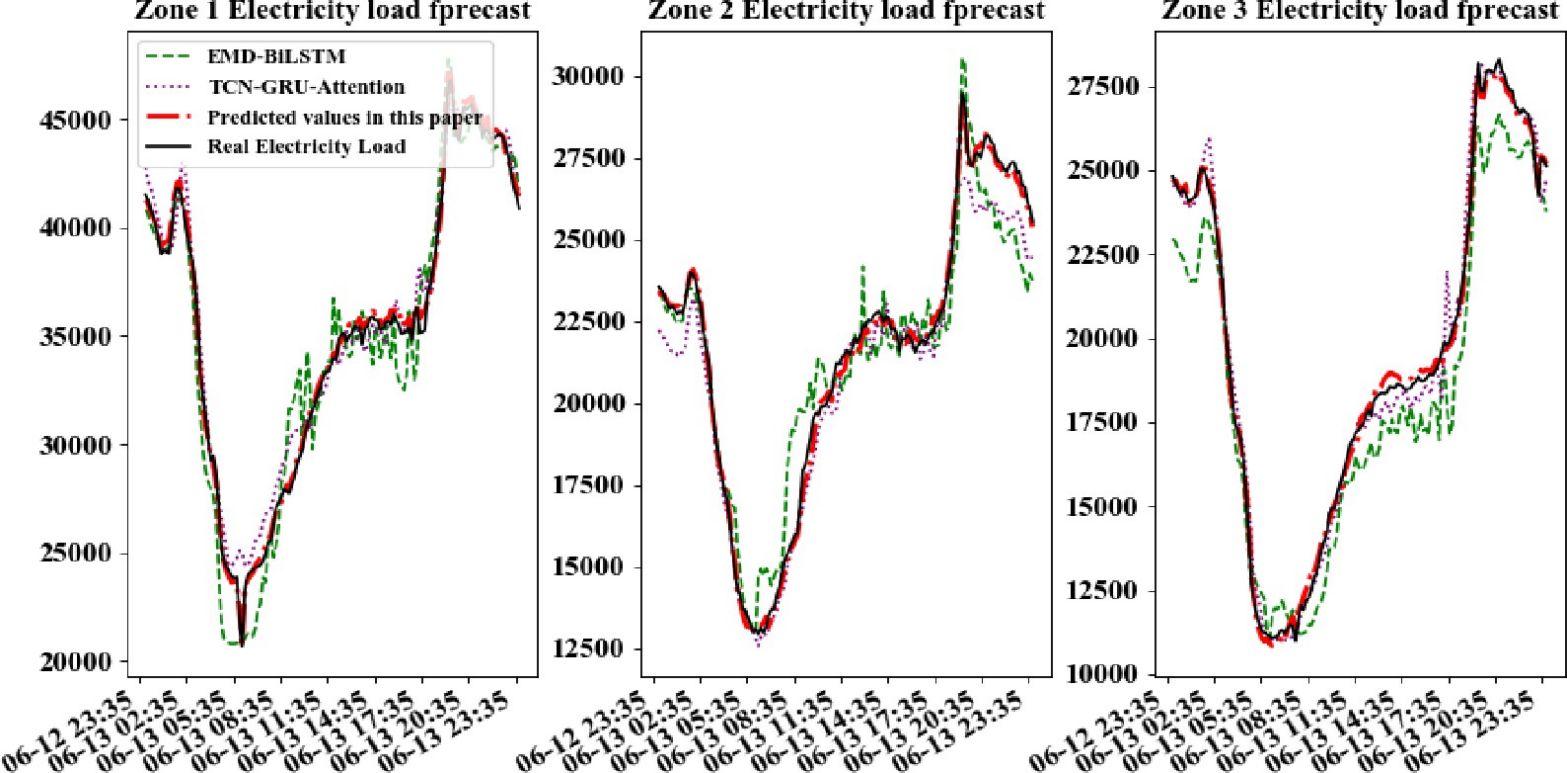
Comparison of forecast model results for a day in the second quarter.

**Fig 9:**
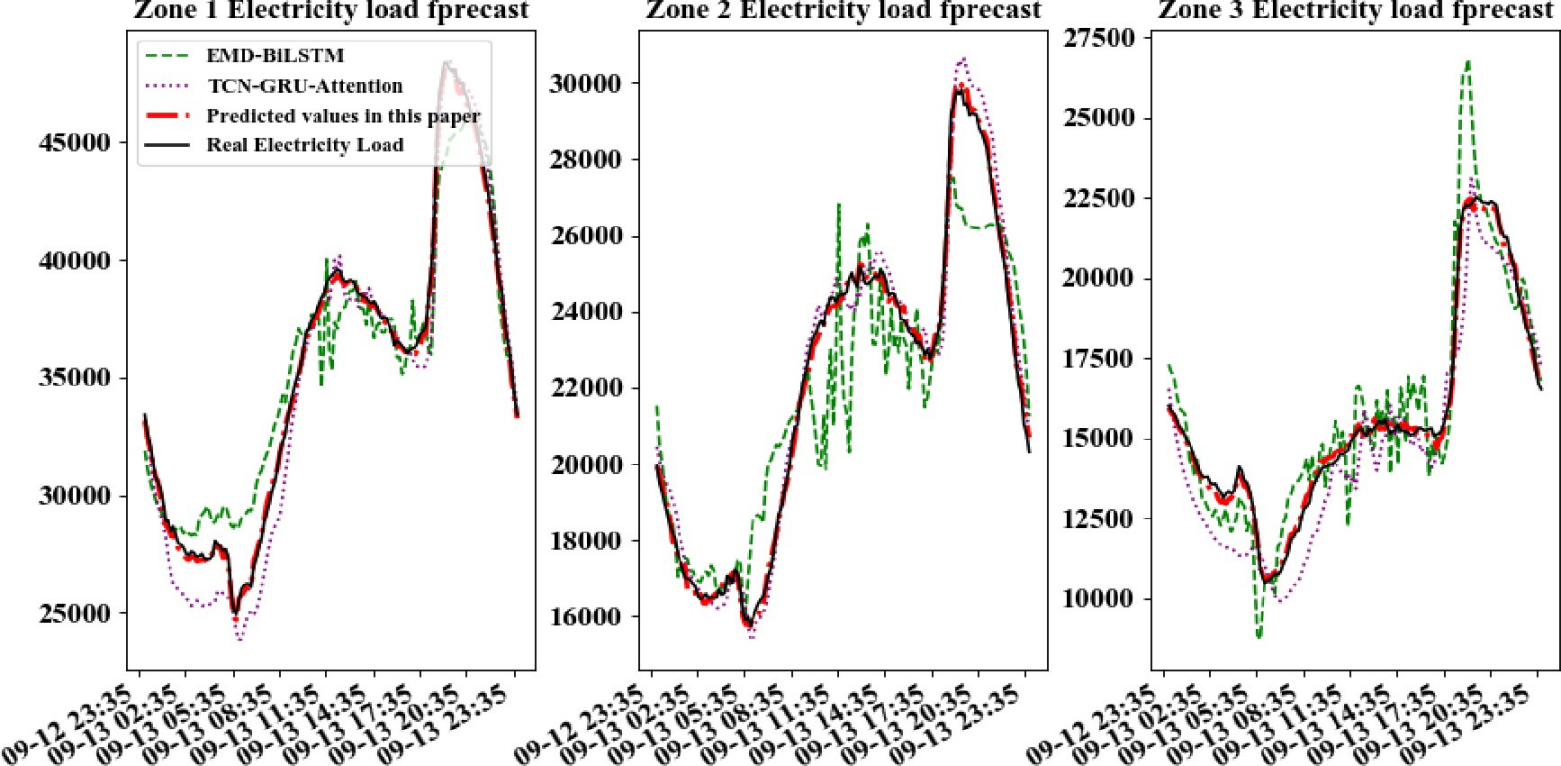
Comparison of forecast model results for a day in the third quarter.

**Fig 10:**
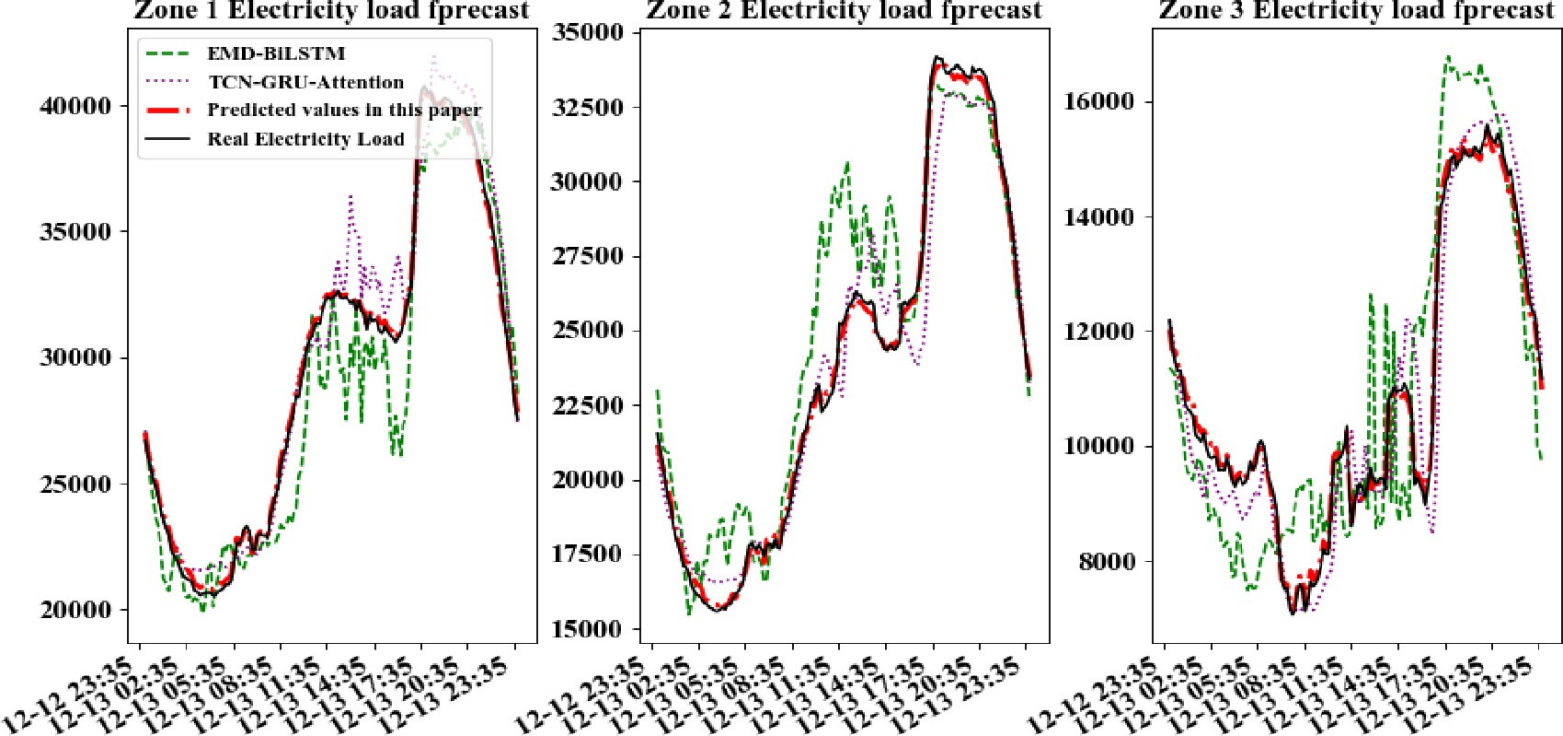
Comparison of forecast model results for a day in the fourth quarter.

In order to further compare the forecastion performance of CD_BiLSTM model with other models more intuitively, electric data of a certain day per quarters is selected randomly in every area for forecast. The practical power load data curves were compared with the forecasted curves, shown in Figs 7–10.

It can be seen from above figures that all models can learn the general trend of electric load change in different quarters. And the model proposed in this paper can fit the practical load change curve to the highest extent. That is, it adapt to the load "spike" change, and learn the trend of electric load change in different quarters. The other comparison models are less able to learn at the "inflection point" which changes rapidly and has less information. So they cannot respond to the rapid change of data in time. At the same time, all models in this paper are required to be multi-output learning structures, which require more powerful learning capability compared to single-output networks. Hence, simple learning structures cannot tap sufficient features to deal with such complex situations. The above comparative analysis shows that the hybrid neural network structure based on sequence-to-sequence framework shows better performance when dealing with multi-output forecasting of ultra-short-term power loads with strong nonlinear relationships.

### 3.4 Robustness analysis

**Fig 11:**
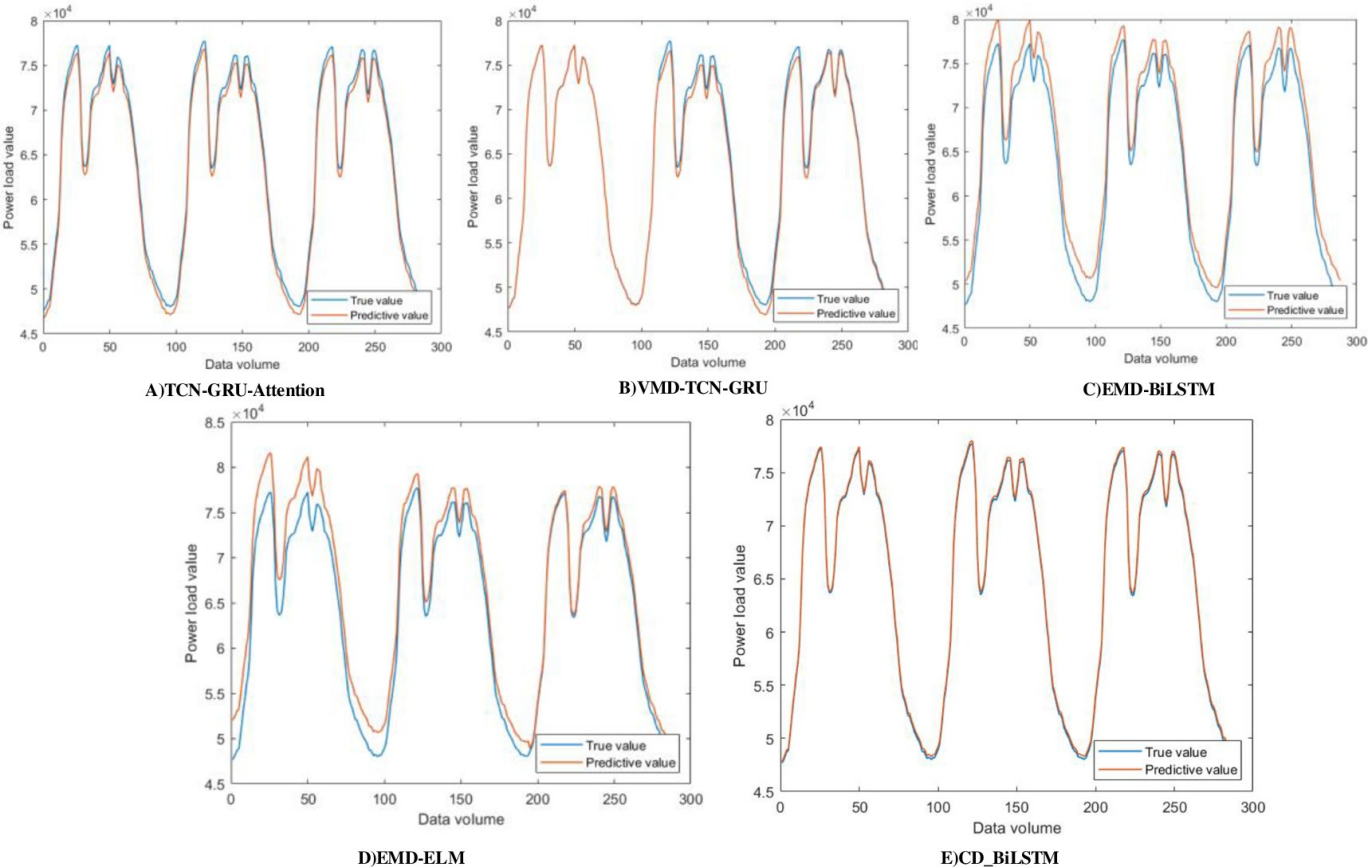
Comparison of other dataset models.

To assess the model’s capacity to make generalizations, we chose a power load dataset from a specific location in southern China. The dataset covers the period from January 1, 2019 to March 31, 2019 and is used to validate the model. Gather data at 15-minute intervals, comprising a grand total of 8640 data points, encompassing multidimensional information on temperature, humidity, wind speed, rainfall, and power load measurements. The training set and test set are partitioned 8:2 in this data set validation experiment, in accordance with the division mode of the model described in this paper. A comparison is made between the CD_BiLSTM model presented in this paper and EMD-ELM, ELM-BiLSTM, VMD-CNN-GRU, and TCN-GRU-Attention in Section 2.3. The effects of the experiments are illustrated in Table 4. To provide a more obvious comparison between different models, three consecutive days of data have been picked due to the substantial volume of data. Fig 11 displays the experimental findings.

**Table 4:** Comparison of prediction effects of different models.

Module	MAE	RMSE
EMD-ELM	2117.2	2519.6
EMD-BiLSTM	1808.1	2012.5
VMD-TCN-GRU	1047.4	1402.4
TCN-GRU-Attention	924.6	1081.6
**CD_BiLSTM**	**367.1**	**628.0**

The information is seen in Table 4 and Fig 11. The model CD_BiLSTM proposed in this paper maintains a good predictive effect, indicating that the model has good generalization performance when applied to other datasets. The adaptability and prediction results of the model to different data sets are satisfactory, and can adapt to the changes of different scenarios.

## 4 Conclusion

In this paper, we propose a hybrid neural network structure based on a sequence-to-sequence framework to mine the feature information that affects the load prediction at the future time. The following conclusions are obtained through experiments:

1. The CD_BiLSTM model constructed in this paper combines the advantages of different neural networks according to the characteristics of electric load data to achieve deep learning of composite features, which can process serial large-sample with robustness, and is suitable to solve ultra-short-term electric load forecasting problems.
2. Due to the obvious differences in power loads under different quarters, we analyze the power loads under different quarters and compared CD_BiLSTM model with 4 learning methods for ultra-short-term power load forecastion. The results show that the model constructed in this paper for multi-output load forecastion under different quarters surpasses the others especially in accuracy.

This paper conducts an initial exploration into the feasibility of constructing a deep learning model through the synchronous learning of power load-related factor characteristics and ontology time sequence information using a framework for sequence-to-sequence learning. The findings of this study offer ideas and technical method methods that can be applied to further research in this field. But the following problems remain with the model: This paper divides the data into four stages according to the quarter, trains the power load forecasting model in different quarters, and less considers the impact of holidays on the power load. On this basis, in the future, the weekend and holiday will be considered as the time criterion to make the power load forecasting scheme more refined.

## Supporting information

S1 File (ZIP)
